# Dietary energy balance modulates ovarian cancer progression and metastasis

**DOI:** 10.18632/oncotarget.2168

**Published:** 2014-07-05

**Authors:** Zaid Al-Wahab, Calvin Tebbe, Jasdeep Chhina, Sajad A. Dar, Robert T. Morris, Rouba Ali-Fehmi, Shailendra Giri, Adnan R. Munkarah, Ramandeep Rattan

**Affiliations:** ^1^ Division of Gynecology Oncology, Wayne State University, Detroit, MI; ^2^ Department of Women's Health, Gynecology Oncology, Henry Ford Hospital, Detroit, MI; ^3^ Department of Pathology, Karmanos Cancer Institute, Wayne State Univeristy, Detroit, MI; ^4^ Department of Neurology, Henry Ford Hospital, Detroit, MI

**Keywords:** ovarian cancer, energy balance, calorie restriction, high energy diet, AMPK, SIRT1, mTOR

## Abstract

A high energy balance, or caloric excess, accounts as a tumor promoting factor, while a negative energy balance via caloric restriction, has been shown to delay cancer progression. The effect of energy balance on ovarian cancer progression was investigated in an isogeneic immunocompetent mouse model of epithelial ovarian cancer kept on a regimen of regular diet, high energy diet (HED) and calorie restricted diet (CRD), prior to inoculating the animals intraperitoneally with the mouse ovarian surface epithelial ID8 cancer cells. Tumor evaluation revealed that mice group on HED displayed the most extensive tumor formation with the highest tumor score at all organ sites (diaphragm, peritoneum, bowel, liver, kidney, spleen), accompanied with increased levels of insulin, leptin, insulin growth factor-1 (IGF-1), monocyte chemoattractant protein-1 (MCP-1), VEGF and interleukin 6 (IL-6). On the other hand, the mice group on CRD exhibited the least tumor burden associated with a significant reduction in levels of insulin, IGF-1, leptin, MCP-1, VEGF and IL-6. Immunohistochemistry analysis of tumors from HED mice showed higher activation of Akt and mTOR with decreased adenosine monophosphate activated kinase (AMPK) and SIRT1 activation, while tumors from the CRD group exhibited the reverse profile. In conclusion, ovarian cancer growth and metastasis occurred more aggressively under HED conditions and was significantly curtailed under CRD. The suggested mechanism involves modulated secretion of growth factors, cytokines and altered regulation of AMPK and SIRT1 that converges on mTOR inhibition. While the role of a high energy state in ovarian cancer has not been confirnmed in the literature, the current findings support investigating the potential impact of diet modulation as adjunct to other anticancer therapies and as possible individualized treatment strategy of epithelial ovarian cancer.

## INTRODUCTION

Ovarian cancer is the fifth leading cause of cancer death in women, making it the most lethal gynecologic cancer [1]. Patients are often diagnosed with advanced stage disease and despite the current treatment of surgical debulking and platinum based chemotherapy, the 5-year survival rate is only 45% [2]. These data highlight the need to identify new approaches that along with the current treatments can assist in bringing about a better outcome for ovarian cancer patients.

Energy balance is defined as the balance of calorie intake and expenditure [3]. An altered energy balance is being associated with pathogenesis of various cancers. A positive energy state, represented by a high body mass index (BMI) or obesity, has been shown to be a risk and a contributing factor in the development of breast [4, 5], prostate [6], endometrial [7], pancreas [8], liver [9], skin [10], colon [11] and other cancers [12]. A negative energy state, achieved by restriction of caloric intake, has been demonstrated to attenuate tumorigenesis in animal models of various cancers [13, 14]. A positive energy balance promotes cancer by creating a tumor promoting environment rich in pro-tumor factors that modify growth signaling, inflammation and angiogenesis, while a negative energy state reduces these changes [15]. The mechanism by which an alerted energy balance leads to modulation of growth and inflammatory factors is still under study. One of the pathways shown to be significantly elevated by high energy diet and reduced by calorie restriction (CR) is the insulin-insulin growth factor (IGF-1) pathway and its downstream signaling leading to the activation of the phosphatidylinositol-3 kinase (PI3K)/Akt- mTOR pathways [14]. High circulating levels of insulin and IGF-1 have been established as risk and prognostic factors for many cancers [16, 17]. The PI3K/Akt pathway, apart from being activated by insulin/IGF-1, integrates signaling from other stimuli and environmental cues to regulate cell survival and proliferation [18]. It is also one of the most commonly activated pathways in all cancers [19]. Hormones like adiponectin and leptin have also been widely shown to undergo alterations under energy modulations [20-22].

The mechanism underlying the shifts in growth factors and hormones brought about by modulation of energy balance is not clear yet. More information is available from the CR models where focus has been on metabolic regulators that may orchestrate the energy dynamics. One of the most studied candidate is the sirtuin family of proteins [23, 24], that has been shown to regulate endocrine signaling, apoptosis and metabolic changes during CR that leads to increased life span [25-27]. A specific role for sirtuins in cancer has not been defined yet, with reports demonstrating sirtuins to have a dual role in the promotion and suppression of tumors [28-30]. Another putative candidate described in mediating CR's benefits is the master metabolism regulator; adenosine monophosphate activated kinase (AMPK). AMPK is a hetero-trimeric serine/threonine protein kinase that acts as an ultra-sensitive cellular energy sensor maintaining the energy balance within the cell [31]. Recently, the role of AMPK in inhibiting proliferation has received attention in tumors of diverse origins [32]. Coupled with its role in inhibiting the protein synthesis in cancer cells [33, 34], controlling gluconeogenesis and glucose uptake and influencing insulin/ IGF-1 levels and signaling [31, 32], makes it an attractive contender mediating the antitumor effects of CR. AMPK and SIRT1 activation is a coordinated occurrence [35], where the downstream inhibition of mTOR is one of the major events [31, 36]. mTOR activation is implicated in almost every tumor type and the process of aging; both of which are slowed by CR, suggesting it to be the central molecule modulated during CR [37].

In this study, we present a comprehensive investigation of how positive and negative energy balance attained by providing high energy diet (HED) and calorie restricted diet (CRD), respectively, modulates ovarian cancer progression in an immunocompetent animal model of ovarian cancer. We provide evidence that a HED accelerates ovarian cancer spread while CR significantly limits it. This is achieved through changes in growth factor and cytokine profiles, which are associated with modulation of AMPK and SIRT1 activation and inhibition of mTOR pathway.

## RESULTS

### Dietary Energy Balance Regulates Ovarian Tumor Growth and Progression

**Figure 1:**
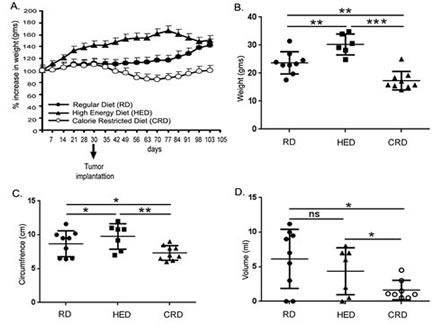
Effect of energy balance on ovarian tumor bearing mice Mouse ovarian tumors were generated by injecting ID8 cells in mice (n = 10) being fed a RD, a HED and a CRD. (A) Average weight progression of mice per group is presented as percentage increase in weight with the average starting weight taken as 100%. (B) Weight at the time of sacrifice (70 days post-tumor injection). (C) Abdominal circumference at the time of sacrifice. (D) Ascites volume as measured after collection at time of sacrifice. ***p < 0.001, **p < 0.01, *p < 0.05, ns = non-significant.

To modulate the energy balance, the 6-week old female C57B6 mice (n = 10) were kept on a dietary regimen of RD, HED and CRD prior to and during the ID8 inoculated ovarian cancer progression as described in the methods. The HED mice gained approximately 40% more average weight than the RD group (Fig. 1A), but towards the end of the study, the HED and RD groups begin to converge, probably due to the accumulating ascites in the RD group and muscle loss in the HED group as the tumor growth progressed. The CRD group had similar average weights to that of the RD mice, until the tumor injections (day 30), after which they experienced a sudden weight loss, which eventually stabilized (Fig. 1A). At the end of the study, the HED mice still had the highest average body weight, while the CRD mice had the lowest (Fig. 1B). The HED mice had the largest abdominal circumference, indicative of tumor and ascites burden, while the CRD mice had significantly less abdominal circumference compared to both RD and especially HED mice (Fig. 1C). The RD and HED mice had a large variation in the volumes of collected ascites. Interestingly, RD mice had a higher average volume of ascites compared to HED mice, although not significant, while the CRD mice consistently had the smallest amount of ascites accumulation (Fig. 1D).

**Figure 2:**
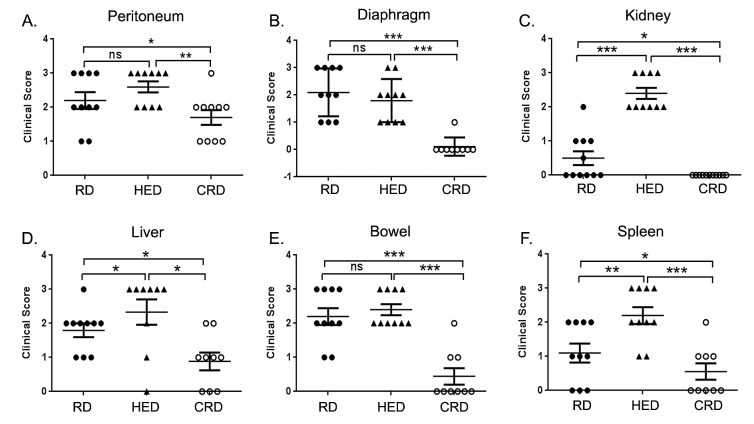
Effect of energy balance on ovarian tumor score At the end of the study, various organs of each mouse from the RD, HED and CRD groups (n = 10), were grossly examined for enumeration of visible tumor nodules. Score was stipulated as 0: no nodule; 1: one nodule; 2: two to five nodules and 3: more than five nodules observed per organ. Tumor scoring at (A) Peritoneum (B) Diaphragm (C) Kidney (D) Liver (E) Bowel (F) Spleen is shown. ***p < 0.001, **p < 0.01, *p < 0.05, ns = non-significant.

**Figure 3:**
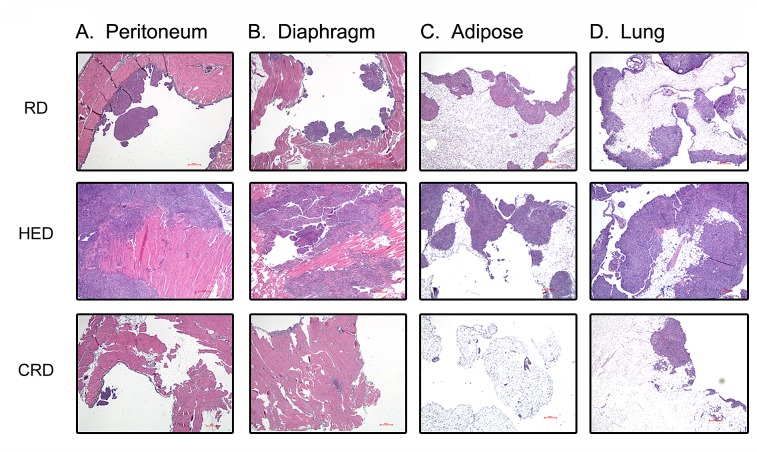
Effect of energy balance on ovarian tumor growth Paraffin tumor sections obtained from the peritoneum (A), diaphragm (B), adipose (C) and lungs (D) were stained with H&E and visualized under a bright-field (200×) to observe for tumor nodules. Each stained tissue picture is a representative of at least 5 individual mouse sections from each of the RD, HED and CRD groups.

Tumor burden was estimated by enumerating the grossly visible tumor nodules on various organs, as described in the methods. The HED mice showed higher tumor burden including the number and size of tumor nodules compared to both RD and CRD groups, while CRD mice showed the least tumor burden. Tumor burden score revealed a significantly higher score in the kidney, liver and spleen of HED mice compared to RD mice (Fig. 2C, D, F). While the tumor score at peritoneum and bowel (Fig. 2A, E) showed a trend towards high but was not significant. The CRD group had a significant decreased tumor score at all sites (peritoneum, diaphragm, kidney, liver, bowel and spleen) compared to the HED and RD group (Fig. 2A-F), except the peritoneum, which did show a decreasing trend but was not statistically significant compared to RD (Fig. 2A). Examination of the H&E stained sections of the organs corroborated the gross tumor score. Sections from HED mice showed the highest number and size of tumor nodules present in the peritoneum, diaphragm, adipose and lung compared to RD or CRD groups (Fig. 3A-D). It was interesting to find tumor nodules metastasized in the lungs, as these nodules were not visible on gross examination (Fig. 3D). The CRD sections from all organs showed the least number and size of tumor growth (Fig. 3A-D). The kidney, liver and spleen H&E sections showed tumor nodules associated on the surface only, and we could not detect any tumors that had invaded the respective tissue (data not shown). Overall, we observed that HED significantly potentiated the ovarian tumor growth, specifically the metastatic spread; however, CRD remarkably reduced the tumor growth and limited the spread of ovarian tumors.

### Dietary Energy Balance Modulated the Levels of Hormone

**Figure 4:**
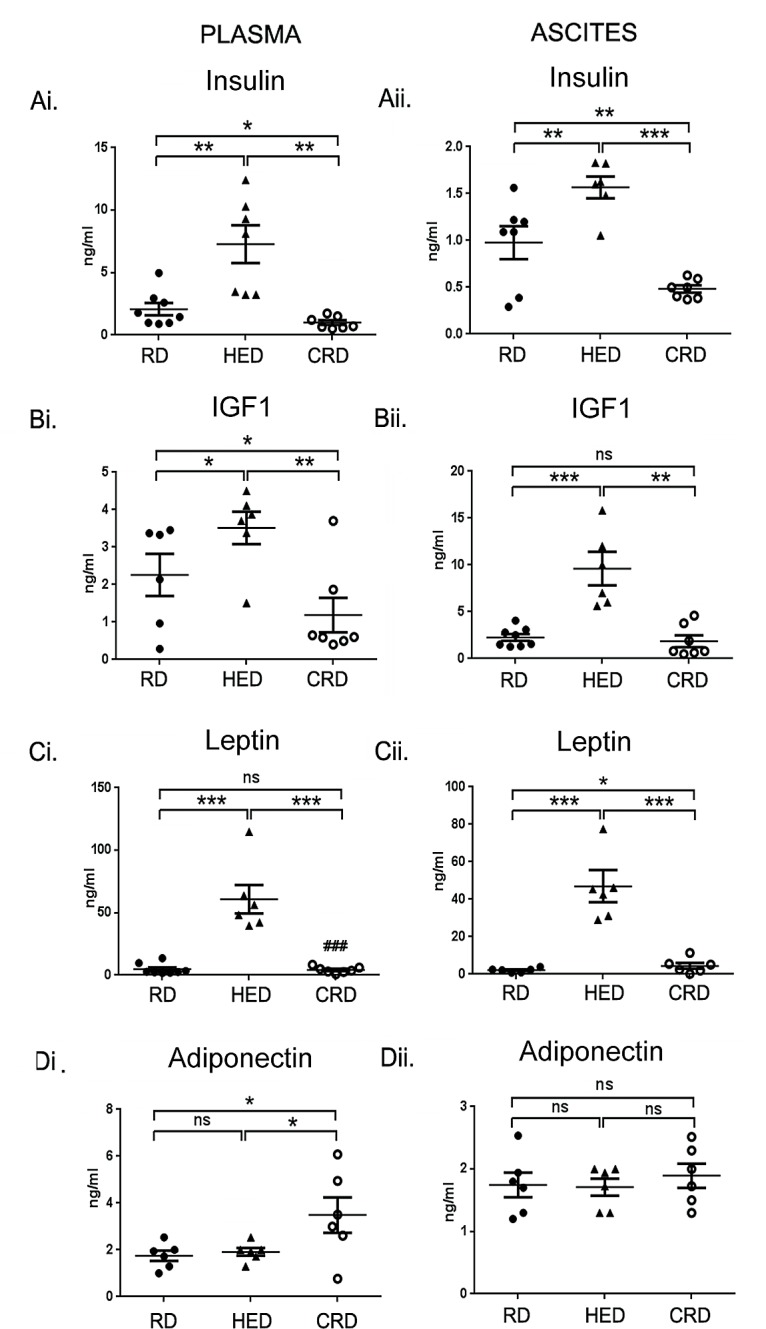
Effect of energy balance on growth factors Plasma and ascitic fluid collected from ovarian tumor mice (n = 6) on RD, HED and CRD at the end of the study (day 70) were subjected to ELISA to determine the levels of (Ai, ii) insulin, (Bi, ii) IGF-1, (Ci, ii) leptin and (Di, ii) adiponectin. ***p < 0.001, **p < 0.01, *p < 0.05, ns= non-significant.

Levels of hormones involved in regulating energy balance including adipokines (leptin and adiponectin), insulin and IGF-1 were estimated in plasma and ascitic fluid by ELISA. The HED mice showed higher levels of insulin, IGF-1 and leptin in both plasma (Fig. 4Ai, Bi, Ci) and ascites (Fig. 4Aii, Bii, Cii), while adiponectin levels were unchanged compared to RD group (Fig. 4Di and ii). CRD mice had the lowest levels of insulin and IGF-1 (Fig. 4Ai and Bi) and increased adiponectin levels in plasma compared to HED and RD groups (Fig. 4Di). In ascites, CRD group had lower levels of insulin, IGF-1 and leptin compared to HED mice (Fig. 4Aii, Bii, Cii), while adiponectin levels were unchanged (Fig. 4Di). Comparing the ascites from CRD and RD groups, significant differences were observed in the levels of insulin and leptin, where insulin levels were lower and leptin was slightly elevated (Figs. 4Aii, Dii). Adiponectin levels did not show any significant alteration among the 3 groups in ascites (Fig. 4Dii). Overall, HED fed mice showed the profile of a tumor promoting environment, while the CRD mice showed an inverse profile, which correlated with the tumor growth seen in the respective groups. Comparing the CRD and RD groups, it can be suggested that the main tumor regressive effects of CRD are associated with decreased production of insulin, IGF-1 and leptin.

### Dietary Energy Balance Modulated the Levels of Cytokines and Angiogenic Factors

**Figure 5:**
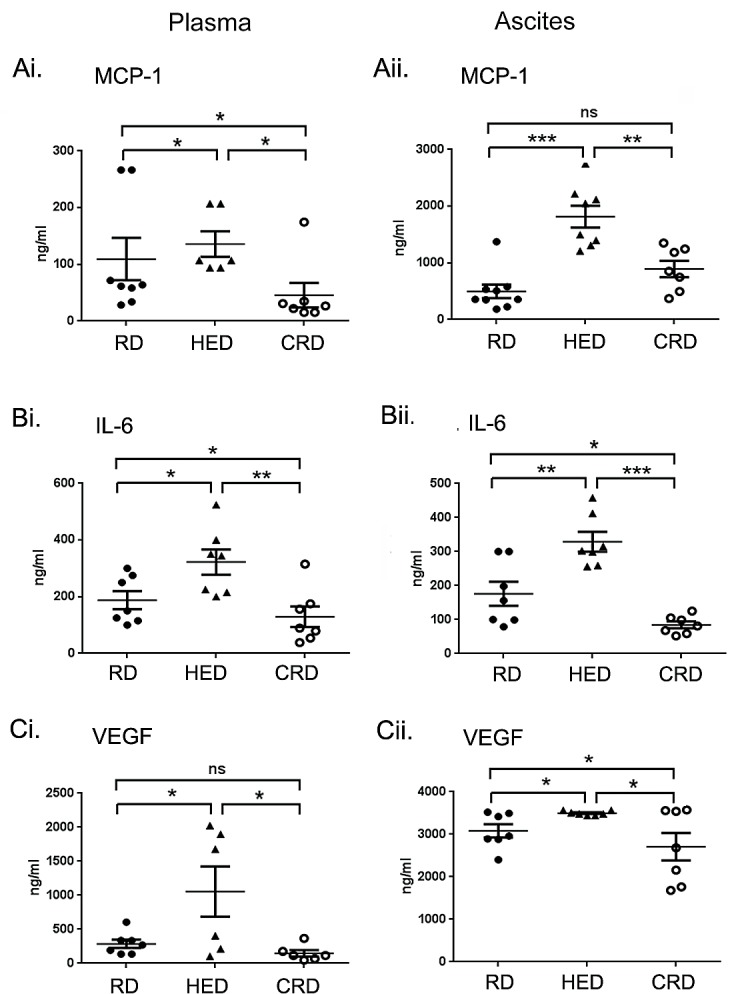
Effect of energy balance on cytokines Plasma and ascitic fluid collected from ovarian tumor mice (n = 6) on RD, HED and CRD at the end of the study (day 70) were subjected to ELISA to determine the levels of (Ai, ii) MCP-1, (Bi, ii) IL-6 and (Ci, ii) VEGF. ***p < 0.001, **p < 0.01, *p < 0.05, ns = non-significant.

Since cytokines (MCP-1 and IL-6) and the angiogenic factor VEGF promote ovarian cancer and are also linked with obesity [38-40], we examined these factors in plasma and ascitic fluids isolated from all groups. The HED group showed an increased production of MCP-1, IL-6 and VEGF in plasma (Fig. 5Ai, Bi, Ci) and ascites (Fig. 5Aii, Bii, Cii), compared to RD and CRD groups. Compared to RD mice, the CRD group had significantly lower levels of MCP-1 and IL-6 in plasma (Fig. Ai, Bi), while VEGF and IL-6 levels were significantly lower in ascites (Fig. 5Bii, Cii). Interestingly, MCP-1 in ascites of CRD mice showed higher (although non-significant) levels than RD group (Fig. 5Aii). These data indicate that diet modulation affects the inflammatory cytokine milieu that may also contribute to changes in the tumor environment with HED, which supports an increased tumor growth, while CR restricts them, resulting in a decreased tumor growth.

### Dietary Energy Balance Modulated the Activation of Akt-mTOR

**Figure 6:**
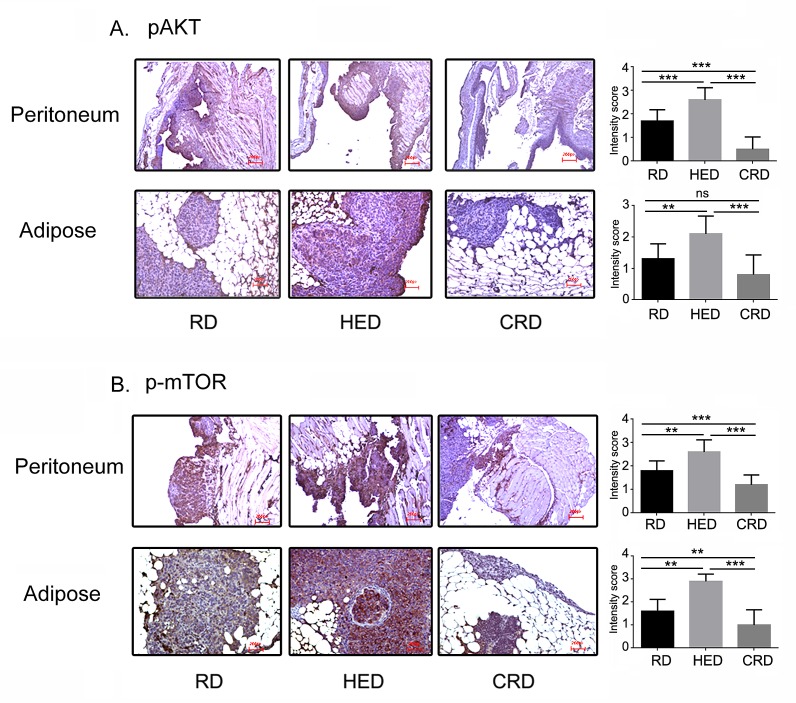
Energy balance modulates Akt- mTOR activation Paraffin tumor sections obtained from the peritoneum and adipose sites of mice from the RD, HED and CRD groups were immunostained with antibodies against phosphorylayed Akt (A) and mTOR (B). Stains were developed using chromogen and visualized under a bright-field (200×) to observe for positive brown stain indicative of expression. Each stained section is a representative of at least 5 different fields examined per section from 3-4 individual stained sections per group.

One of the most established factors altered by energy balance, insulin and IGF-1 converge to activate the Akt-mTOR pathway. Immunohistochemistry analysis revealed that tumor sections from peritoneum and adipose sites of HED group showed higher phosphorylation of Akt (pAkt) and phosphorylated mTOR (pmTOR) (Fig. 6A, B middle panel) compared to RD and CRD groups. The CRD mice tumors from either site had the lowest expression of pAkt and pmTOR (Fig. 6A, B last panel). The quantification of the staining intensity (0-1: no or weak stain; 2: moderate stain and 3: strong stain). The altered phosphorylation of Akt and mTOR in HED and CRD tumors correlated with the corresponding levels of insulin and IGF-1 and the tumor growth observed in the respective groups.

### Dietary Energy Balance Altered the Activation of AMPK and SIRT1

**Figure 7:**
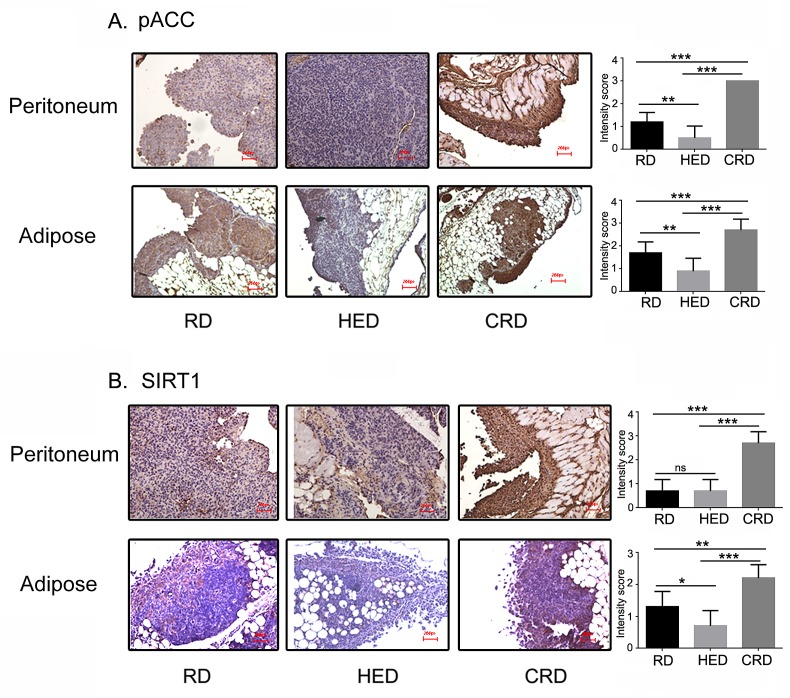
Energy balance modulates AMPK-SIRT1 activation Paraffin tumor sections obtained from the peritoneum and adipose sites of mice from the RD, HED and CRD groups were immunostained with antibodies against phosphorylated ACC, as a marker for AMPK activation (A) and SIRT1 (B). Stains were developed using chromogen and visualized under a bright-field (200×) to observe for positive brown stain indicative of expression. Each stained tissue picture is a representative of at least 5 different fields examined per section from 3-4 individual stained sections per group.

While the modulated levels of insulin, IGF-1, Akt and mTOR in response to energy balance are well reported, the mechanism behind their upstream regulation is not yet defined. To get an insight into the main regulators of energy modulation, we investigated the expression of SIRT1 and AMPK, two energy controlled enzymes associated with the beneficial effects of CR. The RD tumors from peritoneum and adipose sites showed basal level of phosphorylated ACC (pACC), an endogenous substrate of AMPK and a surrogate marker of AMPK activation (Fig. 7A, first panel). The HED tumors showed almost no phosphorylation of ACC, while the CRD tumors showed robust activation of AMPK (Fig. 7A, second and last panels) both in peritoneum and adipose tumors. A similar pattern was observed in the case of SIRT1 (Fig. 7B). Thus, both AMPK and SIRT1, the two energy regulated enzymes are modulated by various dietary conditions in the ovarian tumors.

## DISCUSSION

Energy status of the host as defined by nutritional excess or shortage is now accepted as a factor that can influence the risk and outcome of various cancers. In the present study, we have examined the effect of a positive and negative energy state by dietary modulation on the progression of ovarian cancer. Our data shows for the first time that a positive energy state attained by a fat rich diet encourages an aggressive ovarian growth pattern, while a negative energy state achieved by a 30% CRD results in a limited and restrained ovarian growth pattern *in vivo*.

A positive energy balance, reflected by a state of high BMI or obesity, has been established as a risk factor and associated with a negative prognosis in cancers such as breast, prostate, uterus and others [4, 5, 12]. Association of obesity with ovarian cancer prognosis or outcome is an unresolved issue [41]. Individual and pooled studies have reported an association of BMI with an increased risk of ovarian cancer, while other studies have shown no such correlation. A recent pooled analysis showed that being overweight or obese is associated with a strong increase in the risk of developing borderline serous and invasive endometrioid cancinomas, an intermediate risk for clear cell, invasive and borderline mucinous cancers, but at no increased risk of invasive serous cancer [42]. Recent animal studies and our recent study advocate a strong role of omental and other adipocytes in promoting ovarian tumor growth and metastasis; suggesting a role for adiposity in regulating ovarian tumor progression [43-45]. While difficulties in chemotherapeutic dosing or comorbidities have been linked with the poorer outcomes seen in obese cancer patients, current evidence largely points that the deregulation of host energy balance and associated changes in host and tumor environment can be factors in promoting tumor growth. These changes affect hormones and growth factors like glucose, leptin, adiponectin, insulin and IGF-1. Our current study is the first to illustrate the detrimental effects of a high energy or high fat diet (HED) on ovarian cancer progression. As in other tumor types [46-49], we found that HED promotes an aggressive growth pattern of ovarian tumor spread in the syngeneic ID8 tumor bearing mice. A high tumor burden was observed at most organ sites in HED mice (kidney, liver, spleen, peritoneum and bowel) along with a wider spread to the abdominal adipose tissue and lungs compared to the RD mice (Figs. 1,2 and 3). The plasma and ascitic fluid of HED mice displayed increased levels of growth, inflammatory and angiogenic factors which provide a tumor promoting environment (Figs. 4 and 5).

Hyperinsulinemia, a characteristic of high energy balance, has been shown to increase the risk of occurrence and progression of breast, colorectal, endometrial, prostate and pancreatic cancers [50-52]. Apart from signaling through the insulin receptor, insulin can also signal via the IGF-1 receptor or the insulin receptor/IGF-1 receptor hybrid receptors. Insulin also increases the activity of IGF-1, by promoting its production by the liver and inhibiting the IGF binding protein-1 [52]. Both insulin and IGF-1 induce proliferation and inhibit apoptosis in various cancer cell lines, including ovarian [53, 54]. High circulating levels of IGF-1 have been implicated as a risk and a poor prognosis factor in cancers of the breast, uterus, prostate and colon [50, 55-57]. In ovarian cancer, multiple reports have shown high levels of IGF-1 in cancer cells and in blood, along with increased expression of its receptor [54, 58, 59]. The collective signaling initiated by both insulin and IGF-1 primarily converges on the activation of the PI3K/Akt pathway, leading to the downstream activation of mTOR [52, 60, 61]. The PI3K/Akt-mTOR nexus is the central regulator of cell growth and the most upregulated pathway observed in ovarian and other cancers [60-62]. The increased expression of pAkt and mTOR in tumor sections of HED mice (Fig. 5) support the upregulation of pro-tumor signaling leading to aggressive tumor growth. Leptin is produced by the adipocytes and regulates appetite control and metabolism via the hypothalamus. A positive energy state is characterized by increased levels of circulating leptin. Higher leptin level has been shown to be present in various tumors and is linked with tumor progression in colon, prostate and breast cancer [63-65]. Leptin has been reported to induce proliferation of ovarian cancer cells *in vitro* and overexpression of leptin receptor has been linked to unfavorable prognosis in ovarian cancer patients [66, 67]. Adiponectin, regulates carbohydrate and lipid metabolism, insulin sensitivity and regulates growth pathways [20, 68]. Decreased adiponectin levels have been reported under high energy conditions and in various malignancies [20, 21, 69]. While leptin was increased in HED mice, we did not see any change in adiponectin levels (Figs. 4C, D). One explanation could be that the changes in adiponectin require high energy state of longer duration. Increased levels of MCP-1, IL-6 and VEGF (Fig. 5) indicated the presence of inflammation that is associated with obesity and cancer [70, 71]. MCP-1 and IL-6 are inflammatory cytokines which have been shown to be increased in both obesity and cancer [38, 72, 73]. VEGF is responsible for proliferation and permeability of endothelial cells to mediate angiogenesis and also facilitates metastatic spread [39, 74]. VEGF levels have been shown to be increased in obese individuals even in the absence of tumor [40]. Thus, overall HED transforms the host environment to being more permissive for ovarian tumor growth and spread by providing increased growth factors and cytokines and their responsive signaling. These conditions appear to hasten not only the growth, but also the metastasis of tumor as evident by significant tumor burden and metastasis at distant sites observed in the HED mice.

At the other end of the energy state, a negative energy balance achieved by controlled CR (20-40%) has been demonstrated in animal models to restrict cancer growth [14, 75]. Recently, a study reported a reduction of ovarian and oviduct cancers in calorie restricted egg laying chickens (75). CR results in reduction of the growth hormones, signaling, inflammation and angiogenesis that is increased in HED conditions [75]. In our study the CR mice had the least amount of tumor burden compared to the HED and RD groups. Compared to RD, the CRD mice had significantly low tumor score at all the organ sites examined (peritoneum, diaphragm, kidney, liver, bowel, spleen) and lung metastasis (Fig. 2), similar to reports where CR has been shown to restrict tumor growth [46, 76, 77]. Most of these studies have attributed the tumor inhibitory effect of CR to the deceased circulating levels of insulin and IGF-1 and inhibition in its subsequent downstream signaling of PI3K/Akt-mTOR. Our observation of decreased insulin, IGF-1 and leptin along with low expression of pAkt and p-mTOR (Figs. 4 and 6), are in agreement with these studies. Decreased levels of MCP-1 and IL-6 (Fig. 5) indicate a reduced inflammatory state that has previously been correlated with CR [75]. Additionally CR mice showed decreased VEGF levels, more significantly in the ascites, in concordance with findings from other studies [78]. High VEGF levels, especially in the ascites, are characteristics of ovarian cancer [79, 80]. Overall our data extends the antitumor effects of CR in ovarian cancer and supports its application as a noninvasive adjunct approach towards management of ovarian cancer.

Both excessive and restricted energy states will involve metabolic adaptations by a body to the changing nutritional availability, probably by virtue of nutrient and energy sensors. One of the most promising candidates that fits the requirements is AMPK [81]. AMPK is an established ultrasensitive energy (nutrient) sensor with the ability to regulate metabolic pathways. AMPK can sense the change in the AMP to ATP ratio due to energy stresses including hypoxia, ischemia, exercise, fasting or low nutrient availability. Once activated, AMPK inhibits all ATP consuming anabolic pathways and promotes ATP releasing catabolic pathways. The connection between AMPK and CR has emerged from studies on CR and longevity in genetic and animal models and involves AMPK regulation of PGC-1α, SIRT1, SIRT2, FOXO and autophagy [81]. In our study we observed pACC, the downstream target of AMPK, and its activity marker to be downregulated in tumors of HED mice, while it was highly upregulated in CR mice compared to RD mice (Fig. 7A). AMPK has been established as an inhibitor of the Akt-mTOR pathway in cancer and other cells. AMPK also regulates gluconeogenesis, adipogenesis, lipogenesis, lipolysis and hepatic insulin function [31, 81, 82]. Combined with the differential modulation of AMPK under different energy states, it can be extrapolated that AMPK may be a vital role player in executing CR's anticancer effects. Another protein family linked to CR is the family of deacetylating sirtuin proteins. Sirtuins play a role in endocrine signaling, energy balance and aging [23, 24]. SIRT1, the most studied member of the family, gained attention when it emerged as the main enzyme to be activated in CR mediated increase in life span studies. SIRT1 has been reported to modulate IGF-1, adiponectin and insulin in various tissues [23]. SIRT1 in cancer appears to have a conflicting role. It has been shown to have both a tumor promoting and a tumor inhibitory role in different cancers [28-30]. While an increased phosphorylation of SIRT1 is found under CR conditions and a decreased activity is found in obesity, it has a limited role in CR's antitumor effect [26]. In our study, SIRT1 had a similar expression pattern as AMPK with increased expression in CR tumors and decreased expression in HED tumors (Fig. 7B. This is not very surprising as AMPK has been shown to activate SIRT1 activity [81]. AMPK and SIRT1 have been shown to work together by coordinating their respective phosphorylation and deacetylating functions to regulate other proteins, like mTOR, PGC1a and FOXO, to bring about the modulations seen under CR conditions.

A focal relationship that establishes itself from the study is the activation of AMPK and SIRT1 along with inhibition of mTOR. mTOR is the central nutrient sensor that coordinates the various upstream signaling and environmental stimuli to regulate cell growth and fate [83]. mTOR is the key molecule that regulates cancer growth and the process of aging both in the presence or absence of CR [37]. Inhibition of mTOR by rapamycin has been extensively shown to extend life span and retard cancer growth in various genetic models that include Rb+/−, p53+/−, p53−/− and Fbwx7−/− [84-87]. These studies underline the pivotal role of mTOR in the process of carcinogenesis irrespective of the tumor type or etiology. Thus it can be extrapolated that the upstream metabolic sensors like AMPK and SIRT1 maybe acting mainly through inhibition of mTOR, which results in deacceleration of ovarian tumor progression. This also emphasizes the connection of cancer and aging as deregulation of same factors come into play in both the processes. Ageing is the most significant independent risk factor for occurrence of almost every type of cancer [88]. Thus more in depth studies are required to understand the interplay of AMPK-SIRT1-mTOR nexus that lies at the intersection of regulating cancer and aging.

Our study of the effectors involved in energy balance has been focused on the changes in the tumor and its immediate environment, while energy regulation is a cooperative process involving major organs of muscle, liver and adipose. AMPK and SIRT1, the two energy sensors and effectors are also known to orchestrate the various metabolic pathways in muscle, liver, adipocytes and brain to achieve balance at the organism level. Thus to gain a complete understanding of the effects of energy modulation on tumor growth, we need to examine the host as a whole. These in-depth investigations will offer insight as to how the host status can influence tumor progression and outcome.

In summary, our study is one of the first to define the effect of energy imbalance on ovarian cancer progression. While the HED regime resulted in an aggressive growth, CR significantly limited the growth and metastatic spread of ovarian cancer in the ID8 mouse model. The inverse expression seen in the growth factors (insulin, IGF-1, leptin), inflammatory cytokines (MCP-1, IL-6) and VEGF in high and low energy states clearly point that these are the candidate effectors during such energy shifts. While the mechanism behind the CR is not yet clear, our study points towards an important role of AMPK-SIRT1-mTOR, which could explain the benefits of CR seen in limiting cancer growth. This study opens new possibilities of translating dietary modulation and CR for the prevention and personalized management of ovarian cancer based on the patient's energy status.

## MATERIALS AND METHODS

### Tissue Culture

ID8 mouse ovarian cancer cells were a gift from Dr. Keith Knutson, (Vaccine & Gene Therapy Institute of Florida, Port Saint Lucie, FL) and were tested for absence of standard mouse pathogen panel. ID8 cells were maintained in Rosewell Park Memorial Institute (RPMI) media containing 10% (v/v) FBS. RPMI was purchased from HyClone-ThermoScientific (Waltham, MA). FBS was purchased from BioAbChem (Ladson, SC).

### Animal Studies

All animal experiments were performed according to an Institutional Animal Care and Use Committee of Henry Ford Health Systems approved protocol. Institutional guidelines for the proper and humane use of animals in research were followed. The facility has been approved by the Association for Assessment and Accreditation of Laboratory Animal Care. C57B6 mice were purchased from Jackson Laboratories (Bar Harbor, ME).

### Mouse diet

The various mouse diets were purchased from Bioserv (Frenchtown, NJ). The purified regular diet (RD) was the commonly used balanced nutritional diet used in research (7.2% fat; 61.6% carbohydrate; 20.5% proteins). The nutritionally balanced HED consisted of 60% kilocalories from fat (35.7% carbohydrate; 20.5% protein), while the CRD was similar to RD but with 30% higher supplementation of essential vitamins and minerals to account for the 30% decrease in intake while maintaining the essential nutrients [76, 77]. The RD and HED were provided ad libitum. To achieve 30% calorie restrictive diet, each mouse was housed individually and RD intake was measured each day for a week. The diet was then replaced with the CR at 90% of its RD intake for 1 week, followed by a sequential weekly decrease to 80% to 70% of its RD intake, at which the mouse was maintained for the rest of the study.

### Tumor generation

Six-week to eight-week old female C57B6 mice were weighed and randomized into 3 (n = 10/group) dietary treatment groups: (1) RD (7.2 kcal% fat) fed ad libitum, (2) HED (60 kcal% fat) fed ad libitum and (3) CRD (30% reduced from normal intake). Mice were weighed twice a week. After 30 days of stipulated diet, 5 × 10^6^ ID-8 mouse ovarian cancer cells in 200 μl PBS were injected into the peritoneal cavity of the mice. The mice were monitored daily for any discomfort and weighed twice a week. The diet regimens were continued for another 60 days, after which the mice were sacrificed and autopsied. Ascitic fluid, blood, tumor tissue and vital organs were collected from each mouse.

### Tumor Score

Tumor nodules morphology and count were identified grossly in various organs. These included the liver, spleen, kidneys, bowel, peritoneum and diaphragm. We developed a scoring system to identify the tumor burden in every organ. A score of 0 was given for no nodule in the organ; 1 for one nodule; 2 for two to five nodules and score 3 for more than five nodules observed per organ. Two individuals performed the scoring in a blinded manner and included a gynecology oncology fellow (ZW).

### ELISA

Blood and ascitic fluid were collected to estimate the levels of leptin, adiponectin, insulin, IGF-1, interleukin 6 (IL-6), VEGF and monocyte chemoattractant protein-1 (MCP-1) by ELISA. The insulin ELISA was purchased from Millipore (Billerica, MA) and rest of the kits were purchased from R&D system (Minneapolis, MN). The ELISAs were carried out as per the manufacturer's instructions.

### Immunohistochemistry

The tumors excised from mice were fixed in 10% paraformaldehyde for 48 hours and paraffin-embedded. Consecutive sections of 4 micron thick were cut and processed for hematoxylin and eosin staining (H&E) and immunohistochemistry for p-ACC (cat. no. 3661, used at 1:100), p-mTOR (cat. No: 2976, used at 1:50), p-Akt (Ser473, cat. No: 4060 used at 1:50), SIRT1 (cat no: 15404, used at 1:100) and Ki-67 (cat. No: M7240, used at 1:100). Antibodies to p-ACC, p-mTOR and p-Akt were from Cell Signaling Technology (Denver, MA). Ki-67 was from Dako (Glostrup, Denmark). SIRT1 was from Santa Cruz Biotech (Santa Cruz, CA). Solutions obtained from Dako Cytomation were used for performing immunostaining. In brief, tissue sections were deparaffinized, unmasked, blocked with avidin-biotin, and incubated with primary antibody overnight. Next day, the reaction was detected by using chromogen according to the manufacturer's instruction (Dako). The positive cells stained brown. The slides were examined under a light microscope, and representative pictures were taken from a minimum of 5 or 6 different slides of each group [89]. The quantification of the stain intensity was performed by assigning a score of 0-1 for no or weak stain; 2 for moderate stain and 3 for strong stain. All slides were examined in a blinded manner by two individuals who included a pathologist (RA).

### Statistical Analysis

Data were statistically analyzed using the Graph Pad Prism software (GraphPad Software Inc, La Jolla, CA) using a combination of t-test and ANOVA methods.
